# Secoisolariciresinol Diglucoside of Flaxseed and Its Metabolites: Biosynthesis and Potential for Nutraceuticals

**DOI:** 10.3389/fgene.2018.00641

**Published:** 2018-12-12

**Authors:** Parfait Kezimana, Alexey A. Dmitriev, Anna V. Kudryavtseva, Elena V. Romanova, Nataliya V. Melnikova

**Affiliations:** ^1^Engelhardt Institute of Molecular Biology, Russian Academy of Sciences, Moscow, Russia; ^2^Department of Agrobiotechnology, Peoples’ Friendship University of Russia (RUDN University), Moscow, Russia

**Keywords:** lignans, flaxseed, glycosyltransferases, *Linum*, pinoresinol-lariciresinol reductase, secoisolariciresinol diglucoside, *UGT74S1*, health benefits

## Abstract

Secoisolariciresinol diglucoside (SDG), found mainly in flaxseed, is one of the essential lignans. SDG, as well as the beneficial fatty acid composition and high fiber content, has made flaxseed an important source of functional food or nutraceutical ingredients. Various studies have shown that SDG offers several health benefits, including protective effects against cardiovascular diseases, diabetes, cancer, and mental stress. These health benefits have been attributed to the antioxidant properties of SDG. Additionally, SDG metabolites, namely mammalian lignans, enterodiol and enterolactone, have shown promising effects against cancer. Therefore, understanding the biosynthetic pathway of SDG and its molecular mechanisms is a key to enable the production of new flaxseed cultivars rich in nutraceutical content. The present review highlights studies on the different health benefits of SDG, as well as lignan biosynthesis in flaxseed and genes involved in the biosynthetic pathway. Since SDG, the predominant lignan in flaxseed, is a glycosylated lignan, we also focus on studies investigating the genes involved in secoisolariciresinol glycosylation. These genes can be used to produce new cultivars with a novel level of glycosylation or lignan composition to maximize the yields of lignans with a therapeutic or protective potential.

## Introduction

Secoisolariciresinol (SECO) diglucoside (SDG) is one of the essential dietary lignans, found in high levels in flaxseed (Frank et al., 2004). Along with α-linolenic acid, lignans, mainly SDG, have made flaxseed derivatives (flax oil and lignan extracts) important sources of functional food or nutraceutical ingredients (Hu et al., 2007; Patel et al., 2012). Although flaxseed possesses beneficial fatty acid composition and high fiber content, the phytoestrogenic, anticarcinogenic, and antiatherogenic effects have been attributed to its lignan content (Muir and Westcott, 2003; Frank et al., 2004; Webb and McCullough, 2005; Carraro et al., 2012). Moreover, ingested SDG is converted into mammalian lignans, enterodiol (END) and enterolactone (ENL), by the gut microflora enzymes (Carraro et al., 2012; Struijs, 2008), and these mammalian lignans have been found to negatively correlate with the incidence of breast cancer (Jenab and Thompson, 1996; Frank et al., 2004; Adolphe et al., 2010).

SDG was isolated from flaxseed by Bakke and Klosterman (1956), and in contrast to free lignans found in most plants, SDG is present in an oligomeric structure, referred to as lignan macromolecule (Bakke and Klosterman, 1956; Muir and Westcott, 2003; Struijs, 2008). Flaxseed is the richest source of SDG; however, it also contains small amounts of other lignans, namely pinoresinol, lariciresinol, and matairesinol (Muir and Westcott, 2003; Struijs, 2008; Adolphe et al., 2010). SDG has two enantiomers (+) and (-), whose distribution varies in different *Linum* species. A study showed that one of the two enantiomers of SDG was predominant in the seed, except for some species, such as *L. elegans* and *L. flavum*, that contained both enantiomers (Muir and Westcott, 2003). The (+) enantiomer was predominant in *L. usitatissimum* (flaxseed); however, the concentration of SDG varied in different varieties (Muir and Westcott, 2003). Although new technologies, such as *in vitro* morphogenesis (Lalaleo et al., 2018b), transformed hairy root culture (Gabr et al., 2018) and other biotechnological systems (Lalaleo et al., 2018a), are being used to increase the content of lignans in *Linum* species, the difference in varieties still affects the health beneficial properties of the lignan fractions (Ezzat et al., 2018). In this review, we highlight studies on the biosynthesis of SDG in flaxseed and its health benefits, as well as the genes involved in this metabolic pathway.

## Lignans From Flaxseed

The main lignan in flaxseed is SDG, which is found in a linked macromolecular structure (lignan macromolecule) (Muir and Westcott, 2003; Suzuki and Umezawa, 2007; Struijs, 2008; Touré and Xueming, 2010; Schmidt et al., 2012). In flaxseed, the hull fraction contains higher amounts of lignans than that in the whole seeds (Oomah and Mazza, 1997; Madhusudhan et al., 2000; Wiesenborn et al., 2003; Struijs et al., 2006), suggesting that lignans are mainly biosynthesized in the hull.

## Biosynthetic Pathways of Flaxseed Lignans

The biosynthesis of lignans in flaxseed involves the following pathways (Umezawa, 2003; Hano et al., 2006; Struijs, 2008; Ghose et al., 2014) (KEGG PATHWAY: map01061):

- the phenylpropanoid pathway
- stereospecific coupling by dirigent proteins
- biosynthesis of dibenzylbutane lignans
- glycosylation of lignans into SDG

## The Phenylpropanoid Pathway

The phenylpropanoid pathway is responsible for the formation of the C6-C3 basic unit of lignans. The first step of this pathway is the deamination of phenylalanine by phenylalanine-ammonia lyase producing cinnamic acid (Struijs, 2008). Cinnamic acid is oxidized to *p-*coumaric acid, which can further be oxidized to caffeic acid. Then, caffeic acid can be metabolized into ferulic acid by caffeic acid *O-*methyltransferase. Ferulic acid is converted into its coenzyme A-activated form, feruloyl-CoA by 4-coumarate:CoA ligase. Feruloyl-CoA is further reduced to coniferaldehyde by cinnamoyl-CoA reductase and finally into coniferyl alcohol by cinnamyl alcohol dehydrogenase and sinapyl alcohol dehydrogenase (Boerjan et al., 2003).

## Stereospecific Coupling by Dirigent Proteins

The formation of a lignan via stereoselective coupling of two coniferyl alcohols was first studied in *Forsythia* species in 1990, when two phenylpropanoid monomers, coniferyl alcohols, were oxidized in the presence of the insoluble fraction of *Forsythia* stem, yielding pinoresinol (Umezawa et al., 1990). Later, a protein purified from the insoluble fraction of *Forsythia* stem was used to catalyze the dimerization of coniferyl alcohols only in the presence of an oxidase and was called a dirigent protein since it is not involved in the reaction (Davin et al., 1997; Corbin et al., 2018). The discovery of the dirigent protein (Davin et al., 1997; Davin and Lewis, 2000) allowed the understanding of phenolic radical coupling: an oxidase forms two radicals from the alcohols, and the dirigent protein aligns the radicals, followed by the stereoselective radical coupling reaction. Of all the C6-C3 units formed by the phenylpropanoid pathway, only coniferyl alcohol is dimerized in a stereospecific way (Davin et al., 1997).

After the formation of pinoresinol, lignan biosynthesis can proceed by two different pathways. In the first pathway, the furan structures are reduced, and dibenzylbutane lignans, such as SECO, are formed. In the second pathway, the furan structures remain intact, and methylenedioxy bridged furanofuran lignans, such as sesamin, are formed (Suzuki and Umezawa, 2007; Struijs, 2008; Schmidt et al., 2012). In flaxseed, the biosynthesis follows the first pathway.

## Biosynthesis of Dibenzylbutane Lignans

Pinoresinol is subsequently converted into lariciresinol and secoisolariciresinol by a bifunctional NADPH-dependent pinoresinol/lariciresinol reductase (PLR) (Ford et al., 2001). PLRs completely convert pinoresinol into lariciresinol first before lariciresinol is converted to SECO (Von Heimendahl et al., 2005; Hemmati et al., 2007). The other lignan found in flaxseed, matairesinol, is formed by secoisolariciresinol dehydrogenase (SDH) (Ford et al., 2001; Xia et al., 2001; Umezawa, 2003; Struijs, 2008). The main lignan in flaxseed is found in a glycosylated form, indicating an additional role of the genes responsible for the glycosylation of SECO. These genes are UDPG: glucosyltransferases (GTs), which glycosylate the C-9 and C-9′ hydroxyl positions of SECO (Ford et al., 2001).

## Glycosylation of Seco

In flaxseed, this represents the last step in the biosynthesis of SDG. Glycosylation is catalyzed by GTs, which are highly divergent, polyphyletic, and belong to a multigene family found in all living organisms (Mackenzie et al., 1997; Barvkar et al., 2012).

Several studies have shown the presence of plant GTs (Ross et al., 2001; Wang and Hou, 2009; Caputi et al., 2012). In plants, they belong to family 1 GTs, known as uridine GTs (UGTs) (Caputi et al., 2012; Fofana et al., 2017b). UGTs catalyze the transfer of UDP-activated sugars to specific acceptor molecules (Jones and Vogt, 2001; Ross et al., 2001; Witte et al., 2009). In plants, they are characterized by a unique, well-conserved PSPG (plant secondary product glycosyltransferases) box, formed by a sequence of 44-amino acid residues (Paquette et al., 2003; Gachon et al., 2005; Barvkar et al., 2012) and a catalytic mechanism that inverts the anomeric configuration of the transferred sugar (Wang and Hou, 2009; Barvkar et al., 2012).

In flax, 137 UGT genes have been identified (Barvkar et al., 2012). *UGT74S1* gene expression was high in the seed coat. Since SDG accumulates in the seed coat (Hano et al., 2006), it was suggested that *UGT74S1* might play a role as SECO GT (Barvkar et al., 2012). Further studies found that two UGT genes, *UGT74S1* and *UGT94H*, exhibited high expression level in the developing seeds, and their expression correlated with that of PLR genes, involved in SECO biosynthesis (Ghose et al., 2014). However, upon reaction of the purified proteins with SECO and UDP-glucose, only *UGT74S1* produced both SECO monoglucoside (SMG) and SDG metabolites, showing that *UGT74S1* is involved in SECO glycosylation to form flax SDG lignan (Ghose et al., 2014).

The uniqueness of *UGT74S1* in controlling SDG formation from SECO was further proven in a study characterizing a mutagenized flax population with mutations in *UGT74S1* and altered lignan glycosylation, which showed that the loss-of-function of SECO glycosylation into SDG was attributable solely to *UGT74S1* mutation (Fofana et al., 2017b). UGT74S1 may be the key enzyme controlling SECO glycosylation in flax, although two closely related genes, *UGT74S4* and *UGT74S3*, may also contribute to a minor extent, where they may play a role in supplying SMG as a substrate to *UGT74S1* for the second glycosylation step (Fofana et al., 2017a). Moreover, several studies have shown that specific amino acids within the PSPG motif might play a role (He et al., 2006; Noguchi et al., 2007; Modolo et al., 2009). Additionally, Ghose et al. (2015) have shown that Trp^355^ and His^352^ are critical amino acids within the PSPG motif and are determinant for *UGT74S1* glycosylating activity (Ghose et al., 2015).

**FIGURE 1:**
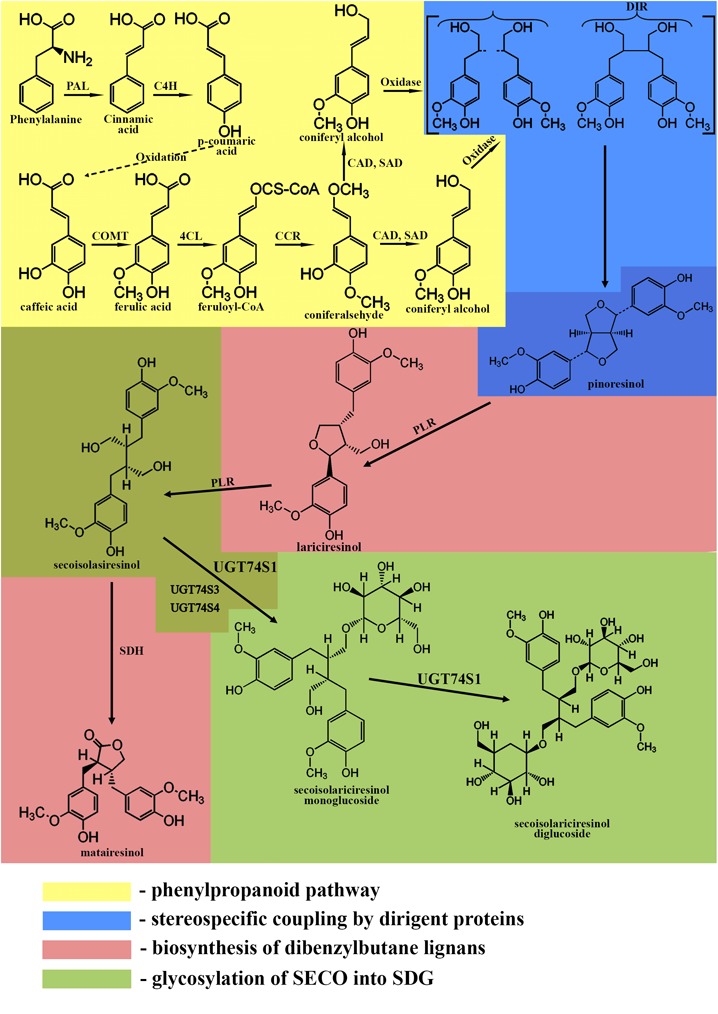
Biosynthetic pathway of SDG. Phenylpropanoid pathway (yellow): PAL, phenylalanine ammonium lyase; C4H, cinnamate 4- hydroxylase; COMT, caffeic acid O-methyltransferase; 4CL, 4-coumarate:CoA ligase; CCR, cinnamoyl-CoA reductase; CAD, cinnamyl alcohol dehydrogenase; SAD, sinapyl alcohol dehydrogenase. Stereospecific coupling (blue); DIR, dirigent proteins. Biosynthesis of dibenzylbutane lignans (pink); PLR, pinoresinol/lariciresinol reductase; SDH, secoisolariciresinol dehydrogenase. Glycosylation of SECO (green); UGT74S1, UGT74S3, UGT74S4, uridine glucosyltransferases (UGTs).

A summary of the biosynthesis of SDG is shown in Figure 1.

## Health Benefits of Sdg

Various studies on the health effects of flaxseed lignans have shown some potential beneficial effects on human and animal health. The conversion of SDG into mammalian lignans, ENL and END, by the intestinal microflora contributes to the bioavailability of SDG; however, other factors, such as diet, antibiotics, and obesity, also affect the circulating lignan levels in the body (Adlercreutz, 2007). Therefore, different levels of lignan bioactivation are observed owing to variations in these factors.

SDG and its mammalian metabolites have been shown to act as nutraceutical agents against some diseases, such as cancer, diabetes, and heart diseases (Touré and Xueming, 2010; Imran et al., 2015). Some of these health effects (Table 1) are discussed in this review.

**Table 1:** Health benefits of secoisolariciresinol diglucoside (SDG) and its metabolites, namely secoisolariciresinol (SECO), enterolactone (ENL), and enterodiol (END).

Health benefits		Reference
Effects on cardiovascular system	• decrease in thrombus formation rate	Sano et al., 2003; Prasad, 2007, 2008, 2009
	• decrease in atherosclerosis	
	• reduction of serum and hepatic cholesterol and LDL-cholesterol levels	Prasad, 2005; Prasad et al., 1998; Fukumitsu et al., 2008; Felmlee et al., 2009
	• increase in vascular endothelial function	Penumathsa et al., 2008
	• suppression or slowing of progression and regression of atherosclerosis	Prasad and Jadhav, 2015
	• protective effects in a monocrotaline-induced model of pulmonary arterial hypertension (PAH)	Puukila et al., 2017
Antidiabetic activity	• reduction of HbA1C in participants with type 2 diabetes	Pan et al., 2007, 2009
	• decrease in insulin and leptin concentrations	Fukumitsu et al., 2008
	• delay in the onset of diabetes by 80%	Prasad, 2001
Anticancer effects	• reduction of mammary tumor incidence	Chen et al., 2003
	• reduction of terminal end bud structures in the mammary gland	Ward et al., 2000; Tan et al., 2004
	• decrease in the expression of COLO 201 human colon cancer cells in athymic mice	Danbara et al., 2005
	• decrease in prostate-specific antigen level and cell proliferation	Demark-Wahnefried et al., 2004
	• suppression of cancer cell proliferation, migration, and metastasis	Chikara et al., 2017; Mali et al., 2017
Effects on mental stress	• reduction of plasma cortisol and small increase in plasma fibrinogen levels during mental stress	Spence et al., 2003; Imran et al., 2015
	• antidepressant-like effect of flaxseed SDG	Ma et al., 2013
Effects on the reproductive system	• reduction of immature ovarian relative weight and delay in puberty	Imran et al., 2015

### Antioxidant Activity of SDG

Some beneficial effects of SDG have been attributed to its antioxidant properties. The ability of SDG to scavenge hydroxyl radicals might contribute to its effects in cancer and lupus nephritis (Prasad, 1997; Touré and Xueming, 2010). SDG and its metabolites have been also shown to prevent DNA oxidative damage and lipid peroxidation (Katare et al., 2012). The antioxidant activities of SECO, SDG, END, and ENL were shown to be involved in the hypocholesterolemic and antiatherogenic effects (Prasad, 2000a,b). Moreover, a recent study comparing flaxseed oil and flaxseed lignan showed that SDG could prevent oxidative stress associated with metabolic syndrome (Pilar et al., 2017).

Additionally, SDG scavenged radiation-induced HOCl in physiological solutions, and a synthetic SDG (LGM2605) was suggested as a promising attenuator of oxidative stress-induced inflammatory tissue damage (Mishra et al., 2018).

### Effects on the Cardiovascular System

Chronic heart diseases, caused directly or indirectly by oxidative stress, inflammation, obesity, diabetes, dyslipidemia, and hypertension, are the leading causes of death in developed nations (Imran et al., 2015). SDG and its metabolites were reported to exhibit cardiovascular protective effects, where they lowered total cholesterol, LDL-cholesterol (LDL-C), and triglyceride levels and normalized HDL-cholesterol (HDL-C) and glucose metabolism, leading to less cardiovascular complications (Zhang W. et al., 2008; Zanwar et al., 2013, 2014). SDG consumption may protect against the development of chronic diseases, such as cardiovascular diseases (Mathieu et al., 2009; O’Keefe et al., 2009; Adolphe et al., 2010).

A recent study on the potential protective effects of SDG in monocrotaline-induced pulmonary arterial hypertension was conducted using male rats. Pretreatment with SDG decreased right ventricular hypertrophy, reactive oxygen species (ROS) levels, lipid peroxidation, catalase, superoxide dismutase, glutathione peroxidase activities, alanine transaminase (ALT), and aspartate transaminase (AST) plasma levels, compared to those in the monocrotaline group. However, cotreatment with SDG did not attenuate ventricular hypertrophy, ALT or AST levels, but decreased ROS levels and catalase and superoxide dismutase activities, compared to those in the monocrotaline group (Puukila et al., 2017).

Secoisolariciresinol diglucoside, even at very low doses (15 mg/kg), suppressed the development of hypercholesterolemic atherosclerosis by 73%, and this effect was associated with a decrease in serum total cholesterol, LDL-C, and oxidative stress, and an increase in HDL-C levels. Therefore, SDG can prevent or delay the progression of atherosclerosis, and hence prevent coronary artery disease, stroke, and peripheral arterial vascular diseases (Prasad and Jadhav, 2015).

### Anticancer Effects

Secoisolariciresinol diglucoside has been shown to prevent some malignancies, such as breast, lung, and colon cancers, owing to its strong antiproliferative, antioxidant, antiestrogenic, and/or antiangiogenic activities. Additionally, the anticancer activity of SDG was suggested to be associated with inhibition of enzymes involved in carcinogenesis (Imran et al., 2015). SDG reduced aberrant crypt multiplicity that may protect against colon cancer (Jenab and Thompson, 1996), and supplementation of SDG in diet resulted in reduction of the volume, area, and number of tumors (Li et al., 1999). In humans, the protective effects of SDG against cancer have been associated with its capability to affect hormone levels and cancer progression (Imran et al., 2015). Furthermore, SDG was considered a chemopreventive agent against malignant mesothelioma owing to its ability to reduce acute asbestos-induced peritoneal inflammation, nitrosative, and oxidative stress (Pietrofesa et al., 2016).

Several studies showed a correlation between SDG metabolites and breast cancer in women, where the serum levels of intestinal microbial END and ENL showed inverse association with breast cancer (Pietinen et al., 2001; Boccardo et al., 2004; Imran et al., 2015). SDG might have the potential to promote early enhancement of mammary gland differentiation, which could protect against breast cancer (Tan et al., 2004; Adolphe et al., 2010). However, García-Mateos et al. (2018) showed that the preventive potential of SDG and its metabolites could be affected by the breast cancer resistance protein (BCRP/ABCG2) (García-Mateos et al., 2018).

Secoisolariciresinol diglucoside was suggested to protect against breast cancer owing to its regulation of the expression level of zinc transporters since the concentration of zinc is higher in breast cancer cells than in the normal breast cells (Zhang L. Y. et al., 2008). Moreover, studies have shown that the anticancer potential of SDG metabolite, ENL, is related to its ability to suppress the proliferation, migration, and metastasis of cancer cells (Chikara et al., 2017; Mali et al., 2017). A research group of Dr. Thompson (University of Toronto) verified the anticancer activity of SDG, where SDG supplementation in mouse diet resulted in a decrease in the tumor load (Shareef and Sarfraz, 2016).

In a clinical trial, the anticancer effects of SDG were studied in 45 premenopausal women with suspicious breast biopsies or who were former breast cancer survivors. SDG lignan (50 mg) was administered daily for a year. Women receiving SDG exhibited less breast precancerous changes, and 80% of them showed a decrease in the levels of Ki-67, a biomarker of cell proliferation (Fabian et al., 2010).

Additionally, the anticancer effects of a purified flaxseed hydrolysate (PFH), a lignan-rich fraction, were studied. PFH of the cultivar Giza9 (rich in SDG) reduced the expression of the metastasis marker, 1-α, metalloproteinases, and vascular endothelial growth factor, one of the most potent stimulators of angiogenesis, whereas it increased caspase-3-dependent apoptosis, suggesting that it exhibited anticancer activity in a human breast cancer cell line T47D. Dietary intake of Giza9 cultivar-derived PFH resulted in a decrease in the tumor volume and an increase in the expression of caspase-3, suggesting that it exerted anticancer activity in tumor-bearing mice (Ezzat et al., 2018).

### Antidiabetic Activity

Apart from flaxseed fibers that may affect insulin secretion and plasma glucose homeostasis, studies have shown that SDG-containing nutrients also affect plasma glucose homeostasis (Touré and Xueming, 2010). SDG reduced C-reactive protein concentration, which is related to insulin resistance in type 2 diabetes (Prasad, 2009; Peterson et al., 2010), and decreased the development of diet-induced obesity (Fukumitsu et al., 2008) and glucosuria (Prasad, 2001). Flax lignan complex decreased the metabolic syndrome composite score in males; however, no effects were observed in females (Cornish et al., 2009). The antidiabetic activity of SDG was suggested to be related to its antioxidant effects (Prasad, 2000b; Prasad et al., 2000).

Secoisolariciresinol diglucoside treatment reduced the incidence of diabetes by 75% in streptozotocin-induced model of diabetes and by 72% in the Bio-Breed diabetic rat model of diabetes. These effects were associated with a decrease in oxidative stress, as evidenced by the decrease in serum and pancreatic malondialdehyde (MDA) levels. SDG was also shown to delay the development of diabetes in Zucker fatty rat type 2 diabetes model, which was associated with reduction of serum MDA and glycated hemoglobin (A1C) levels (Prasad and Dhar, 2015). Thus, SDG may have antidiabetic potential, where it can reduce the incidence of type 1 diabetes and delay the development of type 2 diabetes in humans.

### Effects on Mental Stress

Administration of SDG inhibited stress-induced behavioral changes, whereas treatment with high doses of SDG reversed chronic stress-induced increase in serum corticosterone and adrenocorticotropic hormone (ACTH) levels. Additionally, the effects of SDG on the behaviors of ovariectomized mice might be related to modulation of the neuroendocrine-immune network and neurotrophin factor expression (Ma et al., 2013). Three flax cultivars with different contents of alpha-linolenic acid and lignans significantly reduced blood pressure during frustrating cognitive task-induced mental stress in postmenopausal women with vascular diseases. The cultivar with the highest lignan and lowest alpha-linolenic acid contents was associated with the minimum increase in peripheral resistance and plasma fibrinogen levels and the maximum reduction of plasma cortisol levels during mental stress (Spence et al., 2003).

### Effects on the Reproductive System

The potential effects of SDG on the reproductive system were studied since lignans were reported to have estrogen agonist or antagonist properties. Flaxseed had no effect on rat pregnancy outcome; however, 10% flaxseed-containing diet lowered the birth weight, compared to that of other treatments. Additionally, it exhibited estrogenic effects, including greater uterine and ovarian relative weights, earlier age and lighter body weight at puberty in female offspring, as well as reduced postnatal weight gain and greater sex gland and prostate relative weights in males. However, 5% flaxseed-containing diet reduced immature ovarian relative weight by 29%, delayed puberty by approximately 5 days, and tended to prolong the diestrus, indicating an antiestrogenic effect. Therefore, flaxseed was shown to affect the development of the reproductive system in offspring; thus, caution should be taken when consuming flaxseed during pregnancy and lactation (Tou et al., 1998). Exposure to lignans during lactation reduced the susceptibility to mammary carcinogenesis later in life without adverse effects on selective reproductive indices in dams or offspring (Chen et al., 2003).

## Conclusion

This review summarized the health beneficial effects of SDG, the most predominant lignan in flaxseed, where it can protect against several diseases, including cardiovascular diseases, cancer, diabetes, and mental stress, and affect the reproductive system. Additionally, we reviewed the biosynthetic pathways of SDG in plants.

## Author Contributions

All authors contributed to the preparation of this review.

## Conflict of Interest Statement

The authors declare that the research was conducted in the absence of any commercial or financial relationships that could be construed as a potential conflict of interest.
